# Comparative Analysis of mRNA and lncRNA Expression Profiles in Testicular Tissue of Sexually Immature and Sexually Mature Mongolian Horses

**DOI:** 10.3390/ani14121717

**Published:** 2024-06-07

**Authors:** Yuanyi Liu, Ming Du, Lei Zhang, Na Wang, Qianqian He, Jialong Cao, Bilig Zhao, Xinyu Li, Bei Li, Gerelchimeg Bou, Yiping Zhao, Manglai Dugarjaviin

**Affiliations:** 1 Key Laboratory of Equus Germplasm Innovation, Ministry of Agriculture and Rural Affairs, Hohhot 010018, China; 13470105913@163.com (Y.L.); 13034784525@163.com (L.Z.); 15804814519@163.com (N.W.); qianqianhe202309@163.com (Q.H.); 17647314067@163.com (J.C.); bilig9@163.com (B.Z.); chinalxy94@163.com (X.L.); mulanlb@163.com (B.L.); gerelchimeg@imau.edu.cn (G.B.); yipingzhao@imau.edu.cn (Y.Z.); 2 Inner Mongolia Key Laboratory of Equine Science Research and Technology Innovation, Inner Mongolia Agricultural University, Hohhot 010018, China; 3 Equus Research Center, Inner Mongolia Agricultural University, Hohhot 010018, China

**Keywords:** Mongolian horse, sexually immature, sexually mature, testis, mRNA, lncRNA

## Abstract

**Simple Summary:**

This study explored how genes control the testicular development and sperm production in Mongolian horses. We examined the testes of sexually immature and sexually mature Mongolian horses, focusing on two types of genes: mRNA and lncRNA. Using advanced technology, we found 16,582 mRNAs and 2128 unknown lncRNAs active in both sexually immature and sexually mature Mongolian horses. And 9217 mRNAs and 2191 unknown lncRNAs behaved differently between the two age groups. Our further tests showed that young horses’ genes mostly affected basic cell structures, while those of adult horses influenced hormones, metabolism, and sperm production. These findings deepen our understanding of horse testicular development and may guide future research.

**Abstract:**

Testicular development and spermatogenesis are tightly regulated by both coding and non-coding genes, with mRNA and lncRNA playing crucial roles in post-transcriptional gene expression regulation. However, there are significant differences in regulatory mechanisms before and after sexual maturity. Nevertheless, the mRNAs and lncRNAs in the testes of Mongolian horses have not been systematically identified. In this study, we first identified the testicular tissues of sexually immature and sexually mature Mongolian horses at the tissue and protein levels, and comprehensively analyzed the expression profiles of mRNA and lncRNA in the testes of 1-year-old (12 months, n = 3) and 10-year-old (n = 3) Mongolian horses using RNA sequencing technology. Through gene expression analysis, we identified 16,582 mRNAs and 2128 unknown lncRNAs that are commonly expressed in both sexually immature and sexually mature Mongolian horses. Meanwhile, 9217 mRNAs (*p* < 0.05) and 2191 unknown lncRNAs (*p* < 0.05) were identified as differentially expressed between the two stages, which were further validated by real-time fluorescent quantitative PCR and analyzed using Gene Ontology (GO) and the Kyoto Encyclopedia of Genes and Genomes (KEGG). The analysis results showed that genes in the sexually immature stage were mainly enriched in terms related to cellular infrastructure, while genes in the sexually mature stage were enriched in terms associated with hormones, metabolism, and spermatogenesis. In summary, the findings of this study provide valuable resources for a deeper understanding of the molecular mechanisms underlying testicular development and spermatogenesis in Mongolian horses and offer new perspectives for future related research.

## 1. Introduction

Male gametes play an indispensable role in the development of mammalian zygotes, originating from the dynamic biological process of continuous spermatogenesis and subsequent differentiation within the seminiferous tubules of the testes [1]. This process is crucial for ensuring the normal development of the zygote and the continuation of life. Spermatogenesis refers to the precisely regulated process by which male germ cells proliferate, differentiate, and eventually generate spermatozoa [2,3]. The complete spermatogenesis process can be roughly divided into three stages: Firstly, spermatogonial stem cells (SSCs) originate from primordial germ cells (PGCs) and proliferate through mitosis, differentiating into spermatocytes to maintain the stem cell population and produce differentiated spermatogonia. Secondly, spermatocytes undergo two consecutive meiotic divisions to form haploid round spermatids, with the prophase of meiosis being particularly complex and critical, including the leptotene, zygotene, pachytene, and diplotene stages. Finally, round spermatids undergo morphological and biochemical changes to form mature elongated spermatozoa [4]. These spermatozoa, carrying either X or Y chromosomes, combine with eggs in the ampulla of the fallopian tubes of female animals to develop into zygotes, marking the beginning of a new life [5]. It is worth noting that various somatic cells such as Sertoli cells and Leydig cells in the testes play a critical role during spermatogenesis, and the entire spermatogenesis process is governed by a complex molecular regulatory mechanism ensuring strict and precise control.

Long non-coding RNAs (lncRNAs) are defined as transcripts exceeding 200 nucleotides in length, distinctly different from mRNAs with open reading frames. The completion of the human genome sequencing (in 2004) revealed that only about 1.2% of the genome encodes proteins, supporting early observations (1960s to 1970s) that most of the genome is non-functional, once considered as “junk DNA” in the transcription process [6,7,8]. However, since the 1990s, studies have gradually shown that the so-called “junk DNA” is not a simple accumulation of random sequences but carries important biological information, and some regions are even highly conserved between humans and mice. Entering the 21st century, with the deepening of human genome annotation, it has been found that our understanding of gene structure (especially exons and introns) is still insufficient [9,10]. In recent years, multiple studies have demonstrated that most lncRNAs have specific biological functions. Particularly in mammalian testes, tissue-specific lncRNAs have been found to regulate several key biological processes, including testicular maturation, sperm motility, sperm capacitation, meiosis, and spermatogenesis [11]. For instance, Hong et al. [12] performed an in vitro expression analysis on 26 randomly selected testis-specific lncRNAs and found that these lncRNAs are specifically expressed in testicular tissue, with up to 88% of their expression associated with spermatogenic cells. Weng et al. [13] predicted target genes for lncRNAs in pig testes at different postnatal developmental stages, revealing that these target genes are enriched in metabolic pathways regulating testicular development and spermatogenesis. Ran et al. [14] sequenced lncRNAs in testicular tissue from 30- and 180-day-old pigs and identified 101 differentially expressed lncRNAs that were significantly enriched in seven key pathways, among them TNF, AMPK, and estrogen. Additionally, Zhao et al. [15] screened 1367 differentially expressed lncRNAs in yak testicular tissue and found that upregulation of *VGLL3* may hinder testicular development, while downregulation of *CRACD* is associated with azoospermia. These discoveries not only deepen our understanding of the role of lncRNAs in reproductive biology but also provide new strategies and directions for treating related diseases in the future. However, compared to other livestock, current research on Mongolian horses is still lagging, especially in systematically identifying lncRNAs as key regulatory factors in biological processes. Given the potential role of lncRNAs in various biological processes, this study aims to delve deeper into the regulatory mechanisms of lncRNAs in the development of Mongolian horse testes.

In summary, sexually immature (1-year-old) and sexually mature (10-year-old) Mongolian horses were selected as subjects in this study, and six high-quality RNA sequencing (RNA-seq) libraries were constructed. The core objective of this research is to comprehensively identify and characterize mRNAs and lncRNAs in the testes of Mongolian horses using RNA-seq technology, as well as to screen for key mRNAs, lncRNAs, and their associated mechanisms closely related to testicular development. This is not only the first study to systematically identify lncRNAs in the testicular tissue of immature and mature Mongolian horses, but our data will also lay a solid foundation for further research on testicular development and spermatogenesis mechanisms in Mongolian horses, providing new perspectives and ideas for research in related fields.

## 2. Materials and Methods

### 2.1. Experimental Animal Sample Collection

Testicular tissue samples from Mongolian horses at the Horse Research Center of Inner Mongolia Agricultural University in Hohhot, China, were collected. We selected horses aged 1 year (n = 3) and 10 years (n = 3). Horse ages were verified through management records and dental examination, a reliable age estimation method [16,17]. Samples were gathered after humane euthanasia, approved by the University’s Animal Welfare and Ethics Committee, adhering to ethical and welfare standards.

### 2.2. Paraffin Sectioning and Hematoxylin and Eosin (H.E.) Staining of Mongolian Horse Testicular Tissue

After being fixed at 4 °C for 24 h (fixative: 4% paraformaldehyde) (Solarbio Science and Technology Co., Ltd., Beijing, China), the testicular tissue of Mongolian horses underwent ethanol gradient dehydration for 6 h, followed by xylene (Kemao Chemical Reagent Co., Ltd., Tianjin, China) clearing. After wax melting and degassing at 68 °C, the tissue was immersed in a mixture of xylene and paraffin (Solarbio Science and Technology Co., Ltd., Beijing, China) for 2 h, then transferred to pure paraffin for another 3 h of immersion, prior to embedding and sectioning. The sections were baked at 37 °C for 5 h and stored for future use. The reserved sections were dewaxed with xylene and hydrated (Solarbio Science and Technology Co., Ltd., Beijing, China) with an ethanol gradient, then stained with hematoxylin and eosin. Subsequently, they were treated with hydrochloric acid-alcohol differentiation solution and bluing solution, followed by rinsing with tap water. After staining, the sections were dehydrated with ethanol and xylene, mounted with neutral balsam, and observed under a microscope (Olympus Sales and Service Co., Ltd., Tokyo, Japan).

### 2.3. Western Blot (WB)

Using an extraction buffer, proteins were extracted from the samples, and then the total protein content in the extract was quantified through a quantitative method. The extract was then separated by electrophoresis to isolate the proteins, which were subsequently blotted onto a specific membrane. The proteins on the blot membrane were then eluted using an elution buffer to remove any non-specific proteins. Finally, the eluted proteins underwent immunoassay detection [18,19]. The information related to antibodies is shown in Table S1, and the unedited and uncropped original WB image is shown in Figure S1.

### 2.4. RNA Extraction and Reverse Transcription

mRNA was extracted via the TRlzol method. Subsequently, the extracted mRNA from tissue samples underwent rigorous reverse transcription utilizing the PrimeScript™ RT Master Mix (Perfect Real Time) kit from TaKaRa Bio Inc. (Dalian, China), as referenced in [20,21] (Table S2).

### 2.5. Real-Time Quantitative PCR

Primers were designed with Primer (5.0) software, using referencing sequences from NCBI (https://www.ncbi.nlm.nih.gov, accessed on 10 January 2024), and they were later synthesized by Sangon Biotech Co., Ltd. (Shanghai, China). For real-time quantitative PCR, glyceraldehyde-3-phosphate dehydrogenase (*GAPDH*) was chosen as the internal reference gene, replicated three times technically. The PCR process was conducted with a fluorescent quantitative PCR detection system (BIO-RAD, Hercules, CA, USA). The relative gene expression was determined using the 2^−∆∆Ct^ method [22,23] (refer to Tables S3 and S4).

### 2.6. RNA Transcriptome Sequencing (RNA-seq)

Ribosomal RNA (rRNA) was removed from the total RNA using a ribosome RNA removal kit (Illumina (China) Scientific Instrument Co., Ltd., Shanghai, China) [24]. The RNA was then fragmented into approximately 300 bp segments using ion interruption. Employing RNA as a template, the initial strand of cDNA was produced by utilizing a 6 base random primer alongside reverse transcriptase. Subsequently, the second strand of cDNA was synthesized by referencing the first strand as a template. Once the library was fully constructed, PCR amplification was utilized to enhance the library fragments. Library fragments were then chosen based on size, specifically aiming for fragments of approximately 450 bp. Subsequently, the library was quality checked using an Agilent 2100 Bioanalyzer (Agilent Technologies, Inc., Waldbronn, Germany), and the total and effective concentrations of the library were detected. Based on the effective concentration of the library and the required data volume, libraries with different index sequences (each sample was labeled with a unique index to distinguish the output data for each sample) were mixed in proportion. The mixed library was uniformly diluted to 2 nM and converted into a single-stranded library through alkaline denaturation. After RNA extraction, purification, and library construction, the libraries underwent paired-end (PE) sequencing using next-generation sequencing (NGS) technology on the Illumina HiSeq sequencing platform (Illumina, Inc., San Diego, CA, USA) [25]. The genomic information used in the experiment is shown in Table S5, and the statistics of the annotation of the reference genome are summarized in Table S6.

### 2.7. Bioinformatic Analysis Process of Long Non-Coding RNA (lncRNA)

Firstly, the raw data were filtered to obtain high-quality sequences (clean data), which were then aligned to the reference genome of the species. Based on the alignment results, the expression level of each gene was calculated. Subsequently, differential expression analysis, enrichment analysis, and clustering analysis were conducted on the samples, and the analysis software and parameter settings are shown in Table S7. The aligned reads were assembled to reconstruct transcriptome sequences, which were then compared with known mRNA and lncRNA transcripts to identify novel lncRNAs. Finally, differential expression analysis, target gene enrichment analysis, and clustering analysis were performed on both known and novel lncRNAs [26,27].

### 2.8. Statistical Analysis of Data

The one-way ANOVA analysis was performed using SPSS 22.0 software. Significance testing was conducted using the *t*-test method, and significant results were denoted using the asterisk annotation system. The results are expressed as “mean ± standard error”, where a single asterisk (*) indicates a significant difference at *p* < 0.05, while a double asterisk (**) signifies a highly significant difference at *p* < 0.01 [28,29,30].

## 3. Results

### 3.1. Morphological Differences and Identification of Testicular Tissue in Sexually Immature and Sexually Mature Mongolian Horses

**Figure 1:**
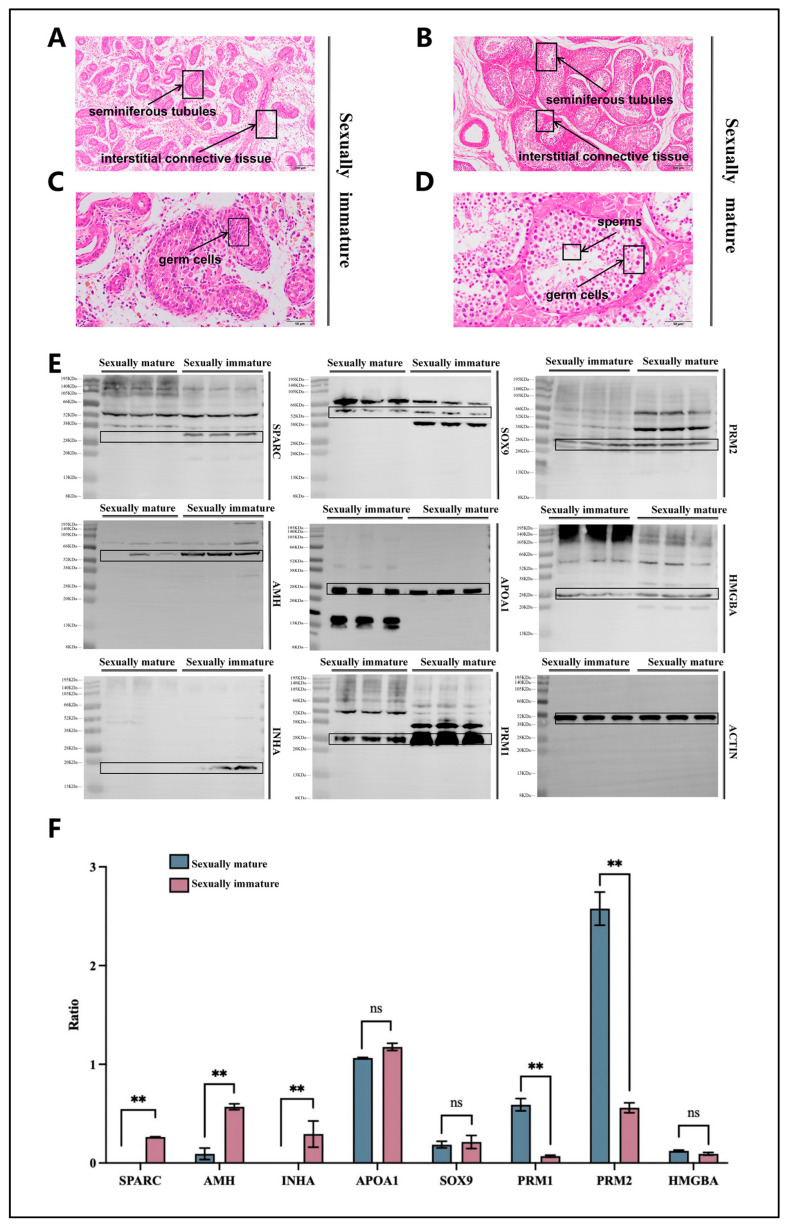
Morphological differences and identification of testicular tissue in sexually immature and sexually mature Mongolian horses. (**A**) Paraffin section and H.E. staining of testis of a 1-year-old Mongolian horse under light microscope at 100× magnification; scale bar 200 μm. (**B**) Paraffin section and H.E. staining of testis of a 10-year-old Mongolian horse under light microscope at 100× magnification; scale bar 200 μm. (**C**) Paraffin section and H.E. staining of testis of a 1-year-old Mongolian horse under light microscope at 400× magnification; scale bar 50 μm. (**D**) Paraffin section and H.E. staining of testis of a 10-year-old Mongolian horse under light microscope at 400× magnification; scale bar 50 μm. (**E**) WB validation of sexually immature, sexually mature marker proteins. (**F**) WB index gray value/inner reference gray value for sexually immature, sexually mature marker proteins (Ratio), ** indicates *p* < 0.01; ns indicates *p* > 0.05.

After conducting an in-depth study on the histological morphology of the testes of Mongolian horses, significant histological differences were observed between the testes of sexually immature and sexually mature horses. Under an optical microscope at 100× magnification, it was clearly visible that the diameter of the seminiferous tubules in the testes of sexually immature Mongolian horses was significantly smaller than that of sexually mature horses (Figure 1A,B). Additionally, differences were also evident in the interstitial connective tissue, which was more developed in the testes of sexually mature horses. To further explore these disparities, we increased the magnification of the microscope to 400×. At this magnification, germ cells in various stages of development were observed in the testes of sexually mature horses, indicating continuous maturation and differentiation of these cells. Importantly, mature spermatids and a small number of free sperms, which are capable of fertilization, were detected in the testes of sexually mature horses. However, no mature germ cells were found in the testes of sexually immature horses (Figure 1C,D).

Meanwhile, to delve deeper into the biological changes before and after sexual maturity in Mongolian horses, we selected marker genes associated with sexual maturity and conducted detailed identification of the testes tissues from both sexually immature and mature horses. In the testes tissues of sexually immature horses, we chose the proteins of SPARC, AMH, INHA, APOA1, and SOX9 marker genes for Western blot (WB) verification. The results showed that the protein expression levels of SPARC, AMH, and INHA were significantly higher in 1-year-old Mongolian horses compared to 10-year-old horses (*p* < 0.01). Although the differences in APOA1 and SOX9 protein expression between the two groups were not statistically significant (*p* > 0.05), the average values still tended to be higher at 1 year old (Figure 1E,F). In the testes tissues of sexually mature horses, we selected the proteins of PRM1, PRM2, and HMGB4 marker genes for WB verification. The results indicated that the protein expression levels of PRM1 and PRM2 were significantly higher in 10-year-old Mongolian horses compared to 1-year-old horses (*p* < 0.01). While the difference in HMGB4 protein expression between the two groups was not significant (*p* > 0.05), the average value showed a trend of being higher at 10 years old (Figure 1E,F).

### 3.2. Sequencing Expression Profiles of mRNA and lncRNA in Sexually Immature and Sexually Mature Mongolian Horse Testes

**Figure 2:**
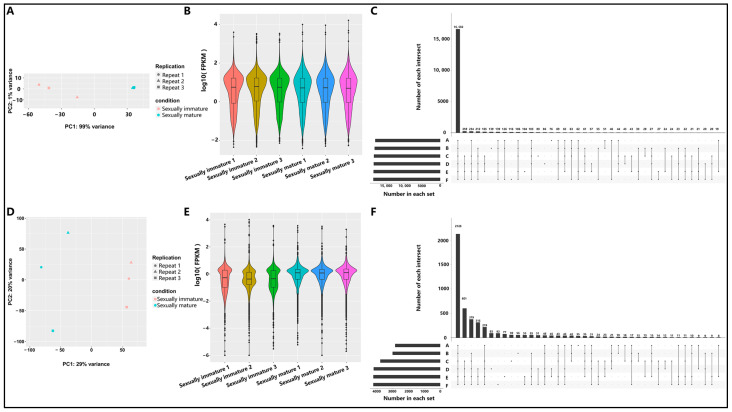
Sequencing expression profiles of mRNA and lncRNA in sexually immature and sexually mature Mongolian horse testes. (**A**) PCA analysis of mRNA. The abscissa represents the first principal component, and the ordinate represents the second principal component. Different shapes in the graph represent different samples, and different colors represent different groups. (**B**) Violin plot of mRNA FPKM. The abscissa represents different samples, while the ordinate represents the logarithm of the FPKM value of the gene to base 10. (**C**) Upset plot of mRNA identified in each sample. “Number in each set” represents the total number of genes identified in each sample; “Number of each intersection” represents the number of genes identified across multiple samples; the connecting line of all points on the abscissa represents the number of common genes identified across all samples, while the remaining single points or connecting lines of multiple points represent the number of unique genes identified in the relevant samples. B: Sexually immature 1, F: Sexually immature 2, A: Sexually immature 3, E: Sexually mature 1, D: Sexually mature 2, C: Sexually mature 3. (**D**) PCA analysis of lncRNA. The abscissa represents the first principal component, and the ordinate represents the second principal component. Different shapes in the graph represent different samples, and different colors represent different groups. (**E**) Violin plot of lncRNA FPKM. The abscissa represents different samples, while the ordinate represents the logarithm of the FPKM value of the gene to base 10. (**F**) Upset plot of lncRNA identified in each sample. “Number in each set” represents the total number of genes identified in each sample; “Number of each intersection” represents the number of genes identified across multiple samples; the connecting line of all points on the abscissa represents the number of common genes identified across all samples, while the remaining single points or connecting lines of multiple points represent the number of unique genes identified in the relevant samples. A: Sexually immature 1, C: Sexually immature 2, B: Sexually immature 3, F: Sexually mature 1, E: Sexually mature 2, D: Sexually mature 3.

By plotting a scatter plot of PC1 and PC2, we conducted an in-depth analysis of the distribution characteristics of mRNA in the testes of sexually immature and sexually mature Mongolian horses. The results showed significant clustering of the three data points for each stage (sexually immature and sexually mature) on the plot (Figure 2A). After normalizing the mRNA data using RPKM, it was found that the overall expression level of the sexually immature Mongolian horse testes group was similar to that of the sexually mature Mongolian horse testes group (Figure 2B). To gain a deeper understanding of mRNA expression, we performed a comprehensive expression analysis of mRNA in Mongolian horse testes. Experimental data revealed that a total of 16,582 mRNAs were detected in multiple samples of both sexually immature and sexually mature horses. Notably, 126 mRNAs were specifically expressed in the testes of sexually immature Mongolian horses, while 165 mRNAs were only expressed in the testes of sexually mature Mongolian horses (Figure 2C).

Regarding the distribution characteristics of lncRNA in the testes of sexually immature and sexually mature Mongolian horses, a scatter plot of PC1 and PC2 was created. The results indicated significant clustering of the three data points for each stage (immature and mature) on the plot (Figure 2D). After normalizing the lncRNA data using RPKM, it was observed that the overall expression level of the sexually immature Mongolian horse testes group was lower than that of the sexually mature group (Figure 2E). To further explore lncRNA expression, a comprehensive expression analysis of lncRNA in Mongolian horse testes was also conducted. The experimental data showed that a total of 2128 lncRNAs were detected in multiple samples from both sexually immature and sexually mature horses. It is worth noting that 42 lncRNAs were specifically expressed in the testes of sexually immature Mongolian horses, while 315 lncRNAs were only expressed in the testes of sexually mature Mongolian horses (Figure 2F). Overall, the expression level of lncRNA was generally lower than that of mRNA (Figure 2C,F).

### 3.3. Differential mRNA Analysis of Testes from Sexually Immature and Sexually Mature Mongolian Horses

**Figure 3:**
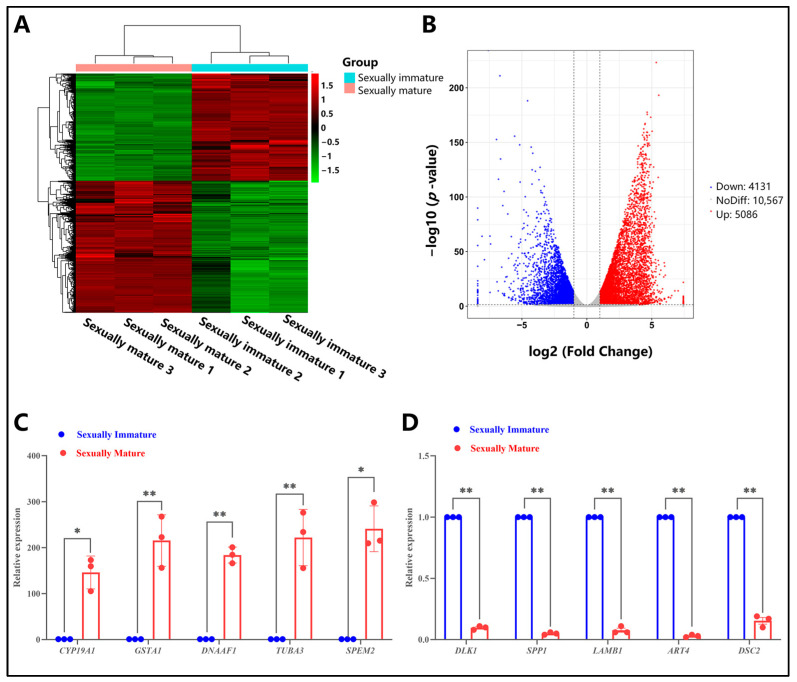
Differential mRNA analysis of testes from sexually immature and sexually mature Mongolian horses. (**A**) Clustering of differentially expressed genes in mRNA. Genes are represented horizontally, with each column representing a sample. Red indicates highly expressed genes, while green indicates lowly expressed genes. (**B**) Volcano plot of differential mRNA expression. The abscissa represents log2FoldChange, while the ordinate represents -log10 (*p*-value). The two vertical dashed lines in the figure represent the threshold for a twofold expression difference; the horizontal dashed line represents the *p*-value = 0.05 threshold. Red dots indicate upregulated genes in the group, blue dots indicate downregulated genes, and gray dots represent non-significantly differentially expressed genes. (**C**) Relative expression of selected screened upregulated differential genes, * indicates *p* < 0.05; ** *p* < 0.01. (**D**) Relative expression of selected screened downregulated differential genes, ** indicates *p* < 0.01.

We conducted differential expression analysis of mRNAs using DESeq, with the criteria for selecting differentially expressed genes set as a fold change of |log2FoldChange| > 1 and a significance level of *p*-value < 0.05. Using the sexually immature age group as the treatment group and the sexually mature age group as the control group, a total of 9217 genes with significant differential expression were identified between the two groups (Figure 3A,B). Compared to the control group, 5086 upregulated genes and 4131 downregulated genes were identified (Figure 3B). To further validate the authenticity of these differentially expressed genes, real-time fluorescent quantitative PCR experiments were performed. The results showed that during sexually maturity, the relative expression levels of *CYP19A1* and *SPEM2* were significantly higher than those in the sexually immature stage (*p* < 0.05); the relative expression levels of *GSTA1*, *DNAAF1*, and *TUBA3* were extremely significantly higher than those in the sexually immature stage (*p* < 0.01) (Figure 3C), whereas the relative expression levels of *DLK1*, *SPP1*, *LAMB1*, *ART4*, and *DSC2* were extremely significantly lower than those in the sexually immature stage (*p* < 0.01) (Figure 3D). This result is highly consistent with the previous sequencing results.

### 3.4. Enrichment Analysis of Differentially Expressed mRNAs in Testes of Sexually Immature and Sexually Mature Mongolian Horses

**Figure 4:**
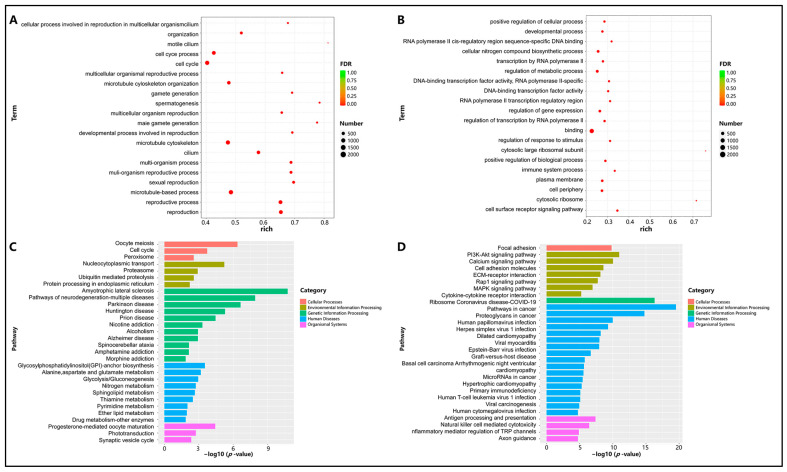
Enrichment analysis of differentially expressed mRNAs in testes of sexually immature and sexually mature Mongolian horses. (**A**) Upregulation of GO terms for differential genes (*p* < 0.01). (**B**) Downregulation of GO terms for differential genes (*p* < 0.01). (**C**) Upregulation of the KEGG pathway for differential gene enrichment (*p* < 0.01). (**D**) Downregulation of the KEGG pathway for differential gene enrichment (*p* < 0.01).

Gene Ontology (GO) enrichment analysis was used to explore the functions of differentially expressed genes in testicular development and the differences between the two gene sets. The upregulated genes were mainly enriched in the following terms: “cellular process involved in reproduction in multicellular organism”, “cilium organization”, “motile cilium”, “cell cycle process”, “cell cycle”, “multicellular organismal reproductive process”, “microtubule cytoskeleton organization”, “gamete generation”, “spermatogenesis”, and “multicellular organism reproduction” (Figure 4A). The downregulated genes were enriched in terms such as “positive regulation of cellular process”; “developmental process”; “cellular nitrogen compound biosynthetic process”; “transcription by RNA polymerase II”; “regulation of metabolic process”; “DNA-binding transcription factor activity, RNA polymerase II-specific”; “DNA-binding transcription factor activity”; and “RNA polymerase II transcription regulatory” (Figure 4B).

Meanwhile, KEGG pathway enrichment analysis was performed on the differentially expressed genes. Among the upregulated genes, approximately 64 pathways related to “Cellular Processes”, “Environmental Information Processing”, “Genetic Information Processing”, “Human Diseases”, and “Organismal Systems” were significantly enriched (*p* < 0.01), such as “Oocyte meiosis”, “Cell cycle”, and “Ubiquitin-mediated proteolysis” (Figure 4C). Among the downregulated genes, 97 significantly enriched pathways were identified (*p* < 0.01), including “Focal adhesion”, “PI3K-Akt signaling pathway”, and “Rap1 signaling pathway” (Figure 4D).

### 3.5. Analysis of Differentially Expressed lncRNAs in Testes of Sexually Immature and Sexually Mature Mongolian Horses

**Figure 5:**
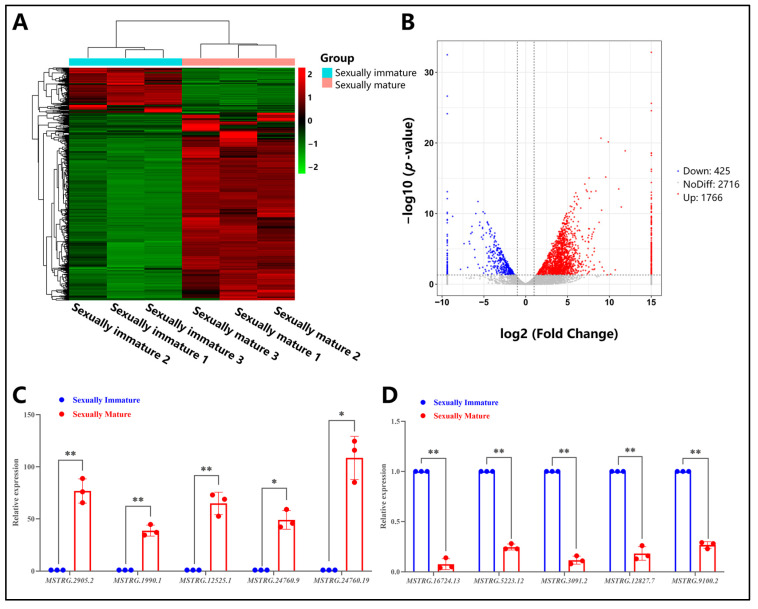
Differential lncRNA analysis of testes from sexually immature and sexually mature Mongolian horses. (**A**) Clustering of differentially expressed genes in lncRNA. Genes are represented horizontally, with each column representing a sample. Red indicates highly expressed genes, while green indicates lowly expressed genes. (**B**) Volcano plot of differential lncRNA expression. The abscissa represents log2FoldChange, while the ordinate represents −log10 (*p*-value). The two vertical dashed lines in the figure represent the threshold for a twofold expression difference; the horizontal dashed line represents the *p*-value = 0.05 threshold. Red dots indicate upregulated genes in the group, blue dots indicate downregulated genes, and gray dots represent non-significantly differentially expressed genes. (**C**) Relative expression of selected screened upregulated differential genes, * indicates *p* < 0.05; ** *p* < 0.01. (**D**) Relative expression of selected screened downregulated differential genes, ** indicates *p* < 0.01.

We conducted differential expression analysis of lncRNAs using DESeq, with criteria for selecting differentially expressed genes set as a fold change of |log2FoldChange| > 1 and a significance level of *p*-value < 0.05. Using the sexually immature age group as the treatment group and the sexually mature age group as the control group, a total of 2191 genes with significant differential expression were identified between the two groups (Figure 5A,B). Compared to the control group, 1766 upregulated genes and 425 downregulated genes were identified (Figure 5B). The number of differentially expressed lncRNAs was relatively smaller compared to mRNAs (Figure 3B and Figure 5B). To further validate the authenticity of these differentially expressed genes, real-time fluorescent quantitative PCR experiments were performed. The results indicate that during the sexually mature age stage, the relative expression levels of *MSTRG.24760.9* and *MSTRG.24760.19* were significantly higher than those in the sexually immature stage (*p* < 0.05). The relative expression levels of *MSTRG.2905.2*, *MSTRG.1990.1*, and *MSTRG.12525.1* were highly significantly elevated compared to the sexually immature stage (*p* < 0.01) (Figure 5C). Conversely, the relative expression levels of *MSTRG.16724.13*, *MSTRG.5223.12*, *MSTRG.3091.2*, *MSTRG.12827.7*, and *MSTRG.9100.2* were significantly lower than those in the sexually immature stage (*p* < 0.01) (Figure 5D). This finding is highly consistent with previous sequencing results.

### 3.6. Analysis of Differential lncRNA Target Genes in Testes of Sexually Immature and Sexually Mature Mongolian Horses

LncRNA sequences are relatively non-conserved, and thus their functions lack conservatism. Different lncRNAs function through various pathways, generally classified into cis-acting and trans-acting mechanisms. Cis-acting target gene prediction assumes that the function of a lncRNA is related to the protein-coding genes located in close proximity to its genomic coordinates. lncRNAs positioned upstream or downstream of protein-coding genes have the potential to intersect with promoters or additional cis-acting elements of concurrently expressed genes, thus influencing gene expression either at the transcriptional or post-transcriptional stage.

**Figure 6:**
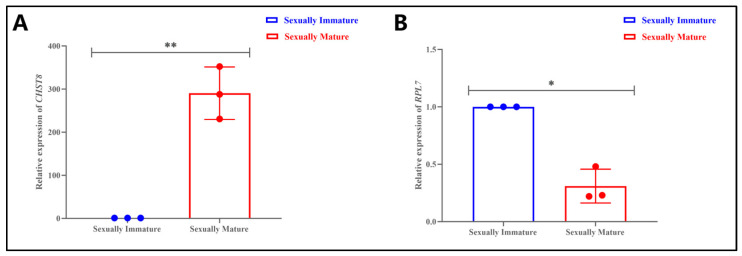
Analysis of differential lncRNA target genes in testes of sexually immature and sexually mature Mongolian horses. (**A**) Relative expression of *CHST8*, ** indicates *p* < 0.01. (**B**) Relative expression of *RPL7*, * indicates *p* < 0.05.

In contrast, the basic principle of trans-acting target gene prediction posits that the function of a lncRNA is independent of its genomic location relative to coding genes and is instead related to co-expressed protein-coding genes. We searched for protein-coding genes within 100 kb upstream and downstream of the lncRNA gene, considering these as potential cis-regulatory target genes for the corresponding lncRNA. Among the top 10 closest positional relationships between the lncRNA and its cis-regulatory target genes, *MSTRG.10053* (target gene: *CHST8*) was selected for relative expression studies using real-time fluorescent quantitative PCR. The results revealed that the relative expression level of *CHST8* was significantly higher in the sexually mature age group compared to the sexually immature age group (*p* < 0.01) (Figure 6A), consistent with the RNA-seq results (Table S8).

The function of trans-regulatory lncRNAs does not depend on their positional relationship with coding genes but is related to co-expressed genes. Trans-target gene prediction is typically performed by calculating the expression correlation between lncRNAs and mRNAs (using the Pearson correlation test) or co-expression analysis. When the sample size is less than 20, a correlation coefficient of |correlation| > 0.9 and a *p*-value < 0.05 are used to screen for trans-acting relationships between lncRNAs and mRNAs. Among the top 10 closest positional relationships between the lncRNA and its trans-regulatory target genes, *MSTRG.24675.2* (target gene: *RPL7*) was chosen for relative expression studies using real-time fluorescent quantitative PCR. The results showed that the relative expression level of *RPL7* was significantly higher in the sexually immature age group compared to the sexually mature age group (*p* < 0.05) (Figure 6B), which is consistent with the RNA-seq results (Table S8).

### 3.7. Enrichment Analysis of Differential lncRNA Target Genes in Testes of Sexually Immature and Sexually Mature Mongolian Horses

**Figure 7:**
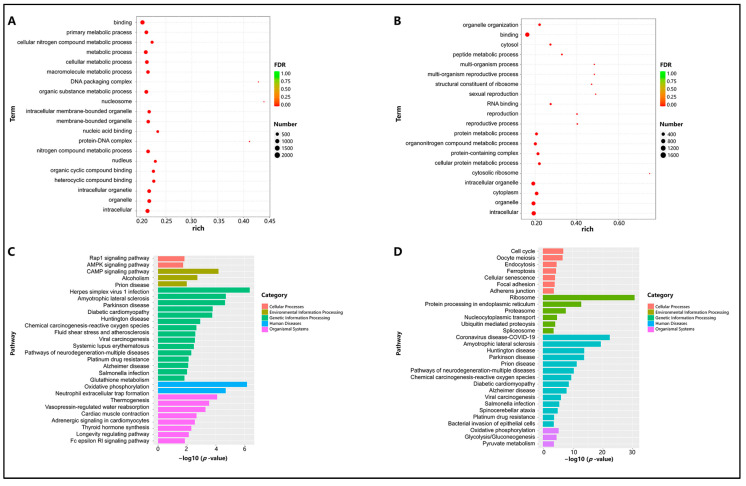
Enrichment analysis of differentially expressed lncRNAs in testes of sexually immature and sexually mature Mongolian horses. (**A**) Upregulation of GO terms for differential genes (*p* < 0.01). (**B**) Downregulation of GO terms for differential genes (*p* < 0.01). (**C**) Upregulation of the KEGG pathway for differential gene enrichment (*p* < 0.01). (**D**) Downregulation of the KEGG pathway for differential gene enrichment (*p* < 0.01).

Gene Ontology (GO) enrichment analysis was conducted to explore the functions of differential genes in testicular development and the differences between the two sets of differential genes. The cis-regulatory genes were mainly enriched in the following terms: “binding”, “primary metabolic process”, “cellular nitrogen compound metabolic process”, “metabolic process”, “cellular metabolic process”, “macromolecule metabolic process”, “DNA packaging complex”, “organic substance metabolic process”, “nucleosome”, and “intracellular-membrane-bounded organelle” (Figure 7A). The trans-regulatory genes were primarily enriched in terms such as “organelle organization”, “RNA binding”, “reproduction”, “reproductive process”, “protein metabolic process”, “cytosolic ribosome”, “intracellular organelle”, and “cytoplasm” (Figure 7B).

Simultaneously, KEGG pathway enrichment analysis was performed on the differentially expressed genes. Among the cis-regulatory genes, approximately 25 pathways related to “Cellular Processes”, “Environmental Information Processing”, “Genetic Information Processing”, “Human Diseases”, and “Organismal Systems” were significantly enriched (*p* < 0.01), including pathways like “Mitophagy-animal”, “Rap1 signaling pathway”, and “AMPK signaling pathway” (Figure 7C). For the trans-regulatory genes, 65 significantly enriched pathways (*p* < 0.01) were identified, such as “Cell cycle”, “Oocyte meiosis”, and “Ferroptosis” (Figure 7D).

## 4. Discussion

### 4.1. Morphological Differences and Identification of Testicular Tissue in Sexually Immature and Sexually Mature Mongolian Horses

Sexual maturity is a critical stage in the developmental process of biological individuals, marking the development and functional perfection of the reproductive system [31,32]. Before sexual maturity, individuals are unable to reproduce, while after sexual maturity, they possess the ability to reproduce [33]. The hallmarks of sexual maturity primarily involve the maturation of gonads and sexual organs, as well as the emergence and development of secondary sexual characteristics [34]. Prior to sexual maturity, the gonads are often in an undeveloped state, whereas after sexual maturity, the gonads undergo a series of changes, such as the ovaries or testes beginning to produce and release mature eggs or sperm [35,36,37]. Through the development of sexual organs, individuals can engage in normal mating and reproductive behaviors. In addition, sexual maturity is accompanied by the emergence and development of secondary sexual characteristics, such as breast development and voice changes. The timing and manner of sexual maturity vary among different organisms. Generally, sexual maturity in animals tends to occur later than in plants, while human sexual maturity is influenced by various factors such as physiology and the environment [38]. The occurrence of sexual maturity is closely related to individual growth, nutrition, endocrinology, and other factors, and it is also influenced by both genetic and environmental factors.

Sexual maturity in animals marks the transition from puberty to full physical and physiological maturity. Therefore, puberty is considered the initial stage of sexual maturity [39]. Puberty is considered a pivotal point in the reproductive process, with a significant role being played in animal sexual development and maturity. The gradual development of the body and reproductive organs leads to individuals being sexually mature. It should be noted that the age of sexual maturity differs among male animals, depending on their species. For example, the age of sexual maturity in pigs is approximately between 5 and 8 months, while in cattle, it is between 10 and 18 months. Horses and donkeys require 18 to 24 months; sheep and goats need 6 to 10 months; camels take 24 to 36 months; and rabbits mature relatively early, needing only 3 to 4 months [40,41,42,43,44,45]. However, as a primitive local breed that gradually formed under natural grazing conditions in alpine regions, the sexual maturation process of the Mongolian horse is influenced by various factors, including the environment and nutrition. Compared to other European and American horse breeds and other livestock, the sexual maturity of Mongolian horses is relatively late, and their development rate is slower [46,47,48]. Studies have shown that male Mongolian horses usually enter puberty around 18 months and reach full sexual maturity after 3 years. Their adult age is generally around 5 years old, and their fertile age can continue until around 15 years old, demonstrating their unique reproductive physiological characteristics [49,50].

In our experiments, we also found that at the age of 1 (12 months), Mongolian horses are not sexually mature. Due to their incompletely developed gonads, the interstitial connective tissue of their testicular tissue is relatively loose compared to that of 10-year-old horses. Simultaneously, the seminiferous tubules in the testes of sexually immature Mongolian horses have a significantly smaller diameter compared to those of sexually mature horses. It is speculated that this delay in sexual maturity may be related to the Mongolian horse’s ability to adapt to alpine environments and resist harsh conditions, as well as its genetic background and physiological mechanisms. According to Liu et al. [51], *APOA1*, *AMH*, *TAC3*, *INHA*, *SPARC*, and *SOX9* were identified as sexually immature marker genes for Mongolian horses due to their specific expression patterns associated with sexual immaturity. These proteins play crucial biological roles in various processes such as cell differentiation, tissue development, and hormonal regulation, which are integral to the sexual immaturity stage [52,53,54,55,56]. On the other hand, *PRM1*, *PRM2*, *H1-9*, *PRSS37*, and *HMGB4* were selected as sexually mature marker genes based on their expression profiles linked to sexual maturity. These proteins are involved in critical functions related to spermatogenesis, chromatin remodeling, and other biological processes essential for sexual maturity [57,58,59,60]. By analyzing the expression of these marker genes, researchers can accurately assess the sexual maturity status of Mongolian horses.

In our study, we selected SPARC, AMH, INHA, APOA1, and SOX9 proteins for WB verification. The results showed that the protein expression levels of SPARC, AMH, and INHA were significantly higher in 1-year-old Mongolian horses than in 10-year-old horses (*p* < 0.01). Although the differences in APOA1 and SOX9 protein expression between the two groups did not reach a significant level (*p* > 0.05), their average values still showed a trend of being higher at 1 year old. We also selected PRM1, PRM2, and HMGB4 proteins for WB verification. The results indicated that the protein expression levels of PRM1 and PRM2 were significantly higher in 10-year-old Mongolian horses than in 1-year-old horses (*p* < 0.01). Although the difference in HMGB4 protein expression between the two groups was not significant (*p* > 0.05), its average value showed a trend of being higher at 10 years old. These results demonstrate significant differences in the testes of Mongolian horses before and after sexual maturity. These differences not only provide important clues for understanding the sexual maturation process of Mongolian horses but also offer valuable references for further studying equine reproductive biology and breeding techniques. Additionally, they identify the samples used in subsequent transcriptome sequencing in this experiment at the protein level.

### 4.2. Differential mRNA Expression and Enrichment Analysis in Testicles of Sexually Immature and Sexually Mature Mongolian Horses

In the sexually immature stage of Mongolian horses, the primary cell type present in their testes is spermatogonia, which are germ cells located within the seminiferous tubules of the testes. These cells possess the ability to proliferate and differentiate. Based on their morphology and developmental stage, spermatogonia can be categorized into different types, including type A spermatogonia (Ad, Ap, and Aal) and type B spermatogonia [61]. As an integral part of the male reproductive system, spermatogonia play a pivotal role in initiating germ cell development, serving as the “seed” cells for spermatogenesis [62,63]. Operating within a complex physiological environment, spermatogonia utilize a self-renewal mechanism to ensure a steady supply of germ cells within the testes [64]. These cells follow a specific gene expression pattern, akin to a cellular “roadmap”, guiding them from their primitive spermatogonia state through development and differentiation into primary spermatocytes [65]. After undergoing a series of complex biological processes, they ultimately transform into mature spermatozoa capable of fertilization. Our experimental results indicate that the relative expression levels of *DLK1*, *SPP1*, *LAMB1*, *ART4*, and *DSC2* are significantly lower in the sexually mature age group compared to the sexually immature age group. The *DLK1* gene encodes a regulatory factor involved in various biological processes such as embryonic development, organ formation, and the proliferation and differentiation of muscle and fat cells [66]. The *SPP1* gene encodes osteopontin, a secreted glycophosphoprotein with functions including cell adhesion, migration, proliferation, and survival [67]. The *ART4* gene encodes a cell surface glycoesterase highly expressed on erythrocyte membranes and involved in immune regulation, apoptosis, and other physiological processes [68]. The *LAMB1* gene encodes the laminin beta-1 chain, a major component of the extracellular matrix that regulates cell adhesion, differentiation, migration, proliferation, and gene expression [69]. The *DSC2* gene, located on chromosome 18, encodes a member of the desmocollin subfamily of cadherins. Democollins and desmogleins are cadherin-like transmembrane glycoproteins that constitute a major component of desmosomes, helping resist shear forces and maintain stable intercellular connections [70]. In summary, these genes highly expressed in sexually immature Mongolian horses are related to fundamental cellular activities such as reproduction, differentiation, and apoptosis. They are speculated to play a crucial role in the proliferation and differentiation of spermatogonia. mRNA enrichment analysis further demonstrates that these genes are primarily enriched in terms such as “positive regulation of cellular process”; “developmental process”; “cellular nitrogen compound biosynthetic process”; “transcription by RNA polymerase II”; “regulation of metabolic process”; “DNA-binding transcription factor activity, RNA polymerase II-specific”; “DNA-binding transcription factor activity”; and “RNA polymerase II transcription regulatory”.

In sexually mature Mongolian horses, sex hormones are crucial for spermatogenesis, stimulating the proliferation and differentiation of germ cells into spermatozoa [71]. Not only do these hormones affect spermatozoa themselves, but they also significantly impact the development and function of reproductive organs, stimulating testicular growth and establishing a conducive environment for spermatogenesis [72,73,74]. The level of sex hormones directly influences sperm quality and quantity, with appropriate hormone levels ensuring normal sperm morphology and viability, thereby enhancing fertilization capability [75]. However, sex hormones do not act alone; they work synergistically with other hormones such as gonadotropin-releasing hormone, follicle-stimulating hormone, and luteinizing hormone to regulate spermatogenesis [76]. *CYP19A1* plays a vital role in this process, encoding aromatase to regulate sex hormone balance in the body, thereby influencing germ cell development and differentiation [77]. Our experiments reveal that the relative expression level of *CYP19A1* is significantly higher in the sexually mature age group compared to the sexually immature age group. As spermatogenesis progresses under the influence of various molecules, cells, and hormones, spermatozoa are ultimately produced [78]. Spermatozoa, the key components of the male reproductive system, carry the important task of transmitting genetic information [79]. Typically composed of a head, neck, and tail, spermatozoa have a highly specialized structure. The head, primarily consisting of a nucleus and acrosome, varies in shape, determined by the morphology of the nucleus and acrosome [80,81,82]. The nucleus of a mature spermatozoon is highly compacted, containing genetic material, while the acrosome holds hydrolytic enzymes crucial for passing through the egg membrane. The primary function of spermatozoa is to fuse with an egg cell, facilitating fertilization [83]. Their motility relies on the structure of their tail, specifically the flagellum [84]. The flagellum comprises an axial filament and a surrounding mitochondrial sheath, enabling effective swimming to locate and bind to an egg cell [85,86]. Genes such as *DNAAF1*, *TUBA3*, and *SPEM2* are closely related to the formation, structure, and function of spermatozoa flagella or cilia, playing a vital role in spermatozoa motility and fertilization capability [87,88,89]. Our experiments show that the relative expression levels of *DNAAF1*, *TUBA3*, and *SPEM2* are significantly higher in the sexually mature age group. Additionally, we found that the relative expression level of *GSTA1*, a gene encoding an enzyme that protects cells from reactive oxygen species and peroxidation products, is also significantly higher in the sexually mature group [90]. This suggests that *GSTA1* may help neutralize reactive oxygen species, protecting the integrity and function of spermatozoa during spermatogenesis. In conclusion, these genes highly expressed during the sexual maturity stage of Mongolian horses play a pivotal role in sexual maturation and spermatogenesis. mRNA enrichment analysis further demonstrates that these genes are primarily enriched in terms such as “cellular process involved in reproduction in multicellular organism”, “cilium organization”, “motile cilium”, “cell cycle process”, “cell cycle”, “multicellular organismal reproductive process”, “microtubule cytoskeleton organization”, “gamete generation”, “spermatogenesis”, and “multicellular organism reproduction”.

### 4.3. Analysis of Differentially Expressed lncRNA Target Genes and Enrichment between Sexually Immature and Sexually Mature Mongolian Horse Testes

The results of cis-regulatory differential gene expression analysis revealed that the relative expression level of CHST8 was significantly higher in sexually mature horses compared to sexually immature ones. The *CHST8* gene encodes a sulfotransferase, which is an important member of the sulfotransferase 2 family with specific biochemical functions [91]. This enzyme is highly expressed in the pituitary gland and is precisely localized to the Golgi membrane, performing the critical task of transferring sulfate to GalNAc residues in glycans [92]. This biochemical process is not only crucial for maintaining normal cellular functions but also involved in a series of complex physiological responses. It is worth noting that the sulfotransferase encoded by the *CHST8* gene is also involved in the sulfation process of luteinizing hormone (LH) [93]. LH is a crucial hormone for sex hormone production, and sex hormones play multiple roles in regulating reproduction, growth, and metabolism in organisms [94]. Therefore, the function of the *CHST8* gene extends beyond biochemical processes to physiological regulation. Cabral et al. [95] have also found that mice lacking the enzyme encoded by the *CHST8* gene exhibit elevated circulating luteinizing hormone levels, which further affects their sexual development, leading to precocious puberty in both male and female mice. This discovery not only reveals the central role of *CHST8* in sex hormone regulation but also suggests that this gene may play a key role in controlling the timing of puberty development. Based on the results of this experiment, we can reasonably speculate that the *CHST8* gene may indirectly affect the process of spermatogenesis in Mongolian horses by finely tuning sex hormone levels. This process includes, but is not limited to, the proliferation, differentiation, and maturation of germ cells, ultimately having a profound impact on sperm production and quality, which is crucial for sexually mature Mongolian horses. Additionally, cis-regulatory gene enrichment analysis showed enrichment in metabolism-related terms such as “binding”, “primary metabolic process”, “cellular nitrogen compound metabolic process”, “metabolic process”, “cellular metabolic process”, “macromolecule metabolic process”, “DNA packaging complex”, “organic substance metabolic process”, and “nucleosome”. Therefore, in subsequent experiments, further investigation into the specific mechanism of the *CHST8* gene in spermatogenesis will help us more fully understand the development and regulation of the reproductive system, providing new ideas and methods for the diagnosis and treatment of related diseases.

The results of trans-regulatory differential gene expression analysis revealed that the relative expression level of *RPL7* was significantly higher in sexually immature horses compared to sexually mature ones. The *RPL7* gene encodes a key component of the ribosomal 60S subunit and plays a crucial role in the growth and development of male animals, especially before sexual maturity [96]. During this stage, male individuals undergo rapid physical growth and organ development, and their demand for protein is particularly high [97]. The *RPL7* gene, through its encoded ribosomal protein, actively participates in the biosynthetic process of proteins, ensuring that all parts of the body receive an adequate and high-quality supply of proteins to support the healthy growth of male animals [98]. In addition, the RPL7 protein exhibits RNA binding activity and transcriptional regulatory ability, both of which play a pivotal role in the regulation of gene expression within cells [99,100]. Based on the results of this experiment, we can reasonably speculate that precise gene expression regulation is crucial for maintaining normal physiological functions and promoting normal morphogenesis during the developmental stage before sexual maturity in Mongolian horses. The *RPL7* gene may participate in this complex regulatory network by affecting RNA stability, transcriptional efficiency, and other means, ensuring that relevant genes can be expressed at the right time and place during various developmental stages. Trans-regulatory gene enrichment analysis also showed enrichment in terms related to cell structure and function, such as “organelle organization”, “RNA binding”, “reproduction”, “reproductive process”, “protein metabolic process”, “cytosolic ribosome”, “intracellular organelle”, and “cytoplasm”. In summary, although we have discovered that the *RPL7* gene plays an important role in male growth and development, the specific mechanism by which the *RPL7* gene directly affects male sexual maturity is not fully understood. Through further research, it is hoped that a more comprehensive understanding of the specific functions and regulatory mechanisms of the RPL7 gene in male physiological development can be attained, offering fresh perspectives and concepts for biological and medical research.

## 5. Conclusions

In summary, this study comprehensively analyzed the mRNA and lncRNA expression profiles of sexually immature and sexually mature testes of Mongolian horses for the first time and compared the differences between the testes of sexually immature and sexually mature Mongolian horses at the tissue and molecular levels, as well as the relevant molecular mechanisms. Furthermore, the relevant lncRNA sequences discovered in this study for the first time also provide a foundation and valuable information for researchers to understand and discover new regulatory mechanisms in the testicular development and spermatogenesis of Mongolian horses.

## Data Availability

All data are available within the article and its Supplementary Materials File or on request from the authors.

## Supplementary Materials

The following supporting information can be downloaded at https://www.mdpi.com/article/10.3390/ani14121717/s1. Table S1: Experimental use of antibody-related information. Table S2: Reverse transcription reaction system. Table S3: Primer information. Table S4: Real-time fluorescence quantitative PCR reaction system. Table S5: Reference genome information. Table S6: Reference genome annotation statistics. Table S7: Analysis software and parameter settings. Table S8: RNA-Seq Map Statistics. Figure S1: Uncropped Western blot figures.
