# Supplementary figures and images for: Comparative Analysis of mRNA and lncRNA Expression Profiles in Testicular Tissue of Sexually Immature and Sexually Mature Mongolian Horses
Source: Animals (Basel). 2024 Jun 7;14(12):1717. doi: 10.3390/ani14121717 (PMC11200857; doi:10.3390/ani14121717)

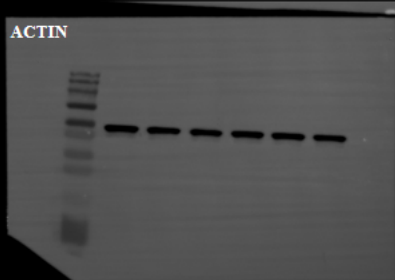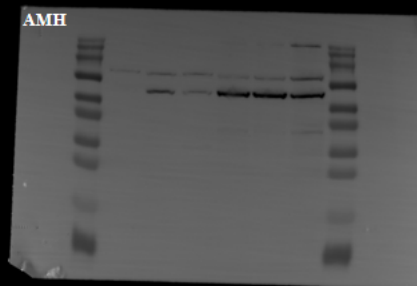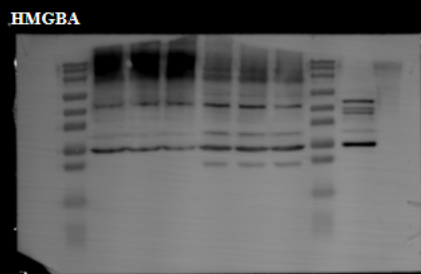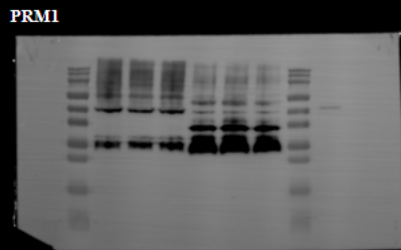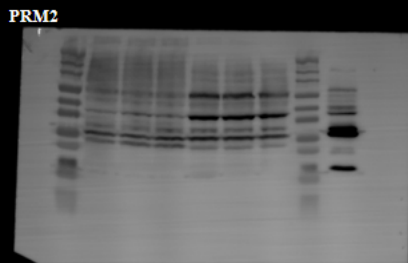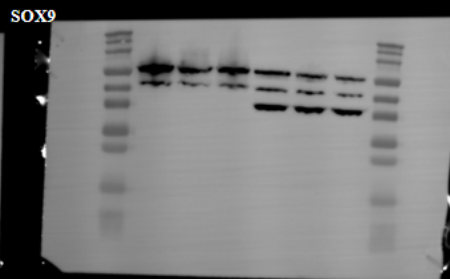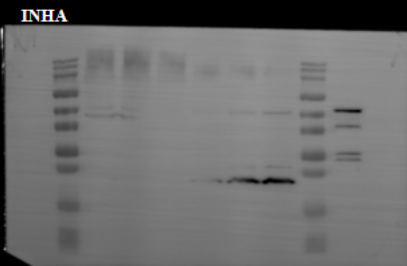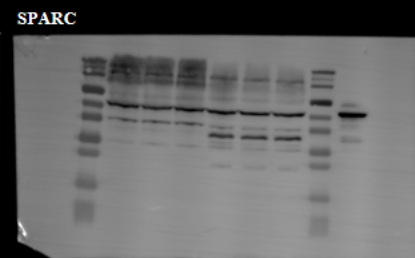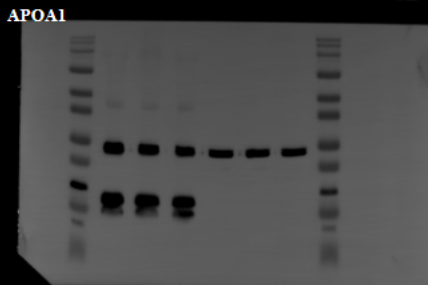

Supplement: Supplementary file 1 [file animals-14-01717-s001.zip › Figure S1 Uncropped Western blot figures.pdf]
